# 4-Fluoro-*N*-(4-hy­droxy­benzyl­idene)aniline

**DOI:** 10.1107/S1600536814015153

**Published:** 2014-07-11

**Authors:** L. Jothi, G. Anuradha, G. Vasuki, R. Ramesh Babu, K. Ramamurthi

**Affiliations:** a Department of Physics, NKR Government Arts College for Women, Namakkal -1, India; b Department of Physics, Kunthavai Naachiar Government Arts College (W) (Autonomous), Thanjavur-7, India; c Crystal Growth and Thin Film Laboratory, School of Physics, Bharathidasan University, Tiruchirappalli 24, India; d Department of Physics and Nanotechnology, Faculty of Engineering and Technology, SRM University, Kattankulathur, Kanchipuram 603 203, India

**Keywords:** crystal structure

## Abstract

In the title compound, C_13_H_10_FNO, the benzene ring planes are inclined at an angle of 50.52 (8)°. A characteristic of aromatic Schiff bases with *N*-aryl substituents is that the terminal phenyl rings are twisted relative to the plane of the HC=N link between them. In this case, the HC=N unit makes dihedral angles of 10.6 (2) and 40.5 (2)° with the hy­droxy­benzene and fluro­benzene rings, respectively. In the crystal, O—H⋯N and C—H⋯F hydrogen bonds lead to the formation of chains along the *c-* and *b-*axis directions, respectively. C—H⋯π contacts link mol­ecules along *a* and these contacts combine to generate a three-dimensional network with mol­ecules stacked along the *b*-axis direction.

## Related literature   

For manufacturing and pharmaceutical applications of Schiff base compounds, see: Akkurt *et al.* (2013 ▶). For related structures, see: Li *et al.* (2008 ▶); Zhang (2010 ▶); Jothi *et al.*, (2012*a* ▶,*b* ▶). For standard bond lengths, see: Allen *et al.* (1987 ▶) and for hydrogen-bond motifs, see: Bernstein *et al.* (1995 ▶).
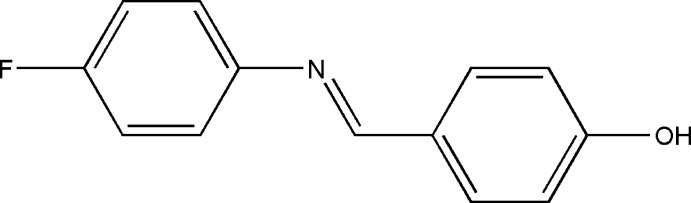


## Experimental   

### 

#### Crystal data  

C_13_H_10_FNO
*M* *_r_* = 215.22
Orthorhombic, 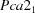

*a* = 11.0153 (8) Å
*b* = 9.8596 (7) Å
*c* = 9.5476 (6) Å
*V* = 1036.93 (12) Å^3^
*Z* = 4
Mo *K*α radiation
μ = 0.10 mm^−1^
*T* = 296 K
0.30 × 0.20 × 0.20 mm

#### Data collection  

Bruker KappaCCD APEXII diffractometer
Absorption correction: multi-scan (*SADABS*; Bruker, 2004 ▶) *T*_min_ = 0.971, *T*_max_ = 0.980
6612 measured reflections
1430 independent reflections
1282 reflections with *I* > 2σ(*I*)
*R*_int_ = 0.033
θ_max_ = 23.4°

#### Refinement  

*R*[*F* ^2^ > 2σ(*F* ^2^)] = 0.029
*wR*(*F* ^2^) = 0.078
*S* = 1.11
1430 reflections
146 parameters
1 restraint
H-atom parameters constrained
Δρ_max_ = 0.13 e Å^−3^
Δρ_min_ = −0.12 e Å^−3^

### 

Data collection: *APEX2* (Bruker, 2004 ▶); cell refinement: *APEX2* and *SAINT-Plus* (Bruker, 2004 ▶); data reduction: *SAINT-Plus* and *XPREP* (Bruker, 2004 ▶); program(s) used to solve structure: *SHELXS97* (Sheldrick, 2008 ▶); program(s) used to refine structure: *SHELXL97* (Sheldrick, 2008 ▶); molecular graphics: *Mercury* (Macrae *et al.*, 2008 ▶); software used to prepare material for publication: *WinGX* (Farrugia, 2012 ▶) and *PLATON* (Spek, 2009 ▶).

## Supplementary Material

Crystal structure: contains datablock(s) I, global. DOI: 10.1107/S1600536814015153/sj5413sup1.cif

Structure factors: contains datablock(s) I. DOI: 10.1107/S1600536814015153/sj5413Isup2.hkl

Supporting information file. DOI: 10.1107/S1600536814015153/sj5413Isup3.cml

CCDC reference: 924015

Additional supporting information:  crystallographic information; 3D view; checkCIF report

## Figures and Tables

**Table 1:** Hydrogen-bond geometry (Å, °) *Cg* is the centroid of the C1–C6 benzene ring.

*D*—H⋯*A*	*D*—H	H⋯*A*	*D*⋯*A*	*D*—H⋯*A*
O1—H1⋯N1^i^	0.82	1.94	2.756 (2)	176
C9—H9⋯F1^ii^	0.93	2.61	3.263 (3)	127
C13—H13⋯*Cg* ^iii^	0.93	2.83	3.710 (3)	157

Symmetry codes: (i) 
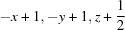
; (ii) 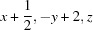; (iii) 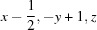.

**Fig. 1.**
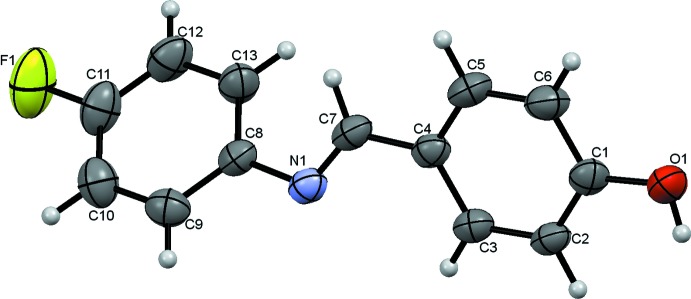
: The molecular structure of the title compound. Displacement ellipsoids are drawn at the 50% probability level.

**Fig. 2.**
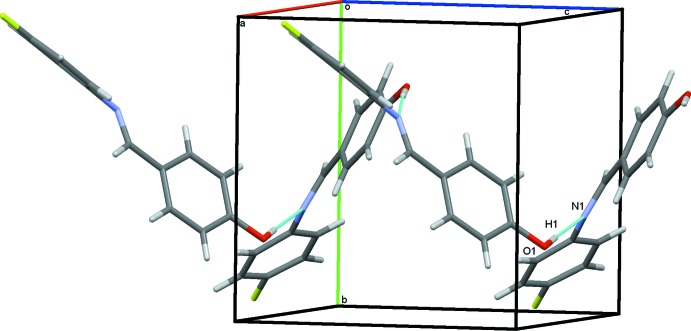
: Chains formed along the c axis by O—H···N hydrogen bonds.

**Fig. 3.**
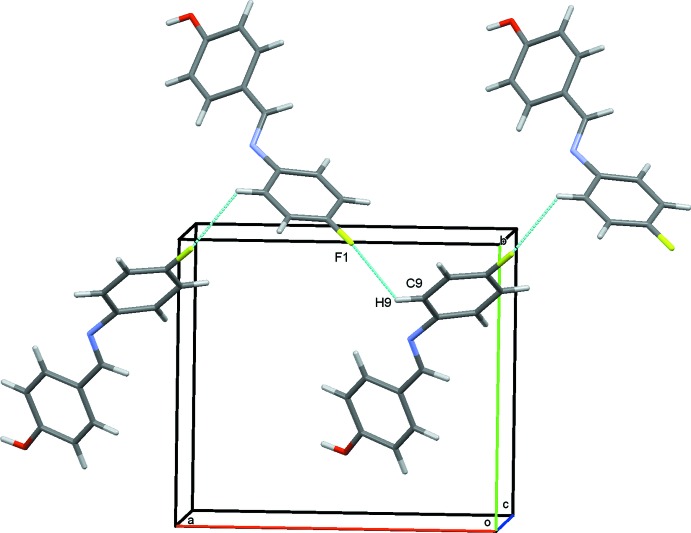
Chains formed along the b axis by C—H···F hydrogen bonds.

**Fig. 4.**
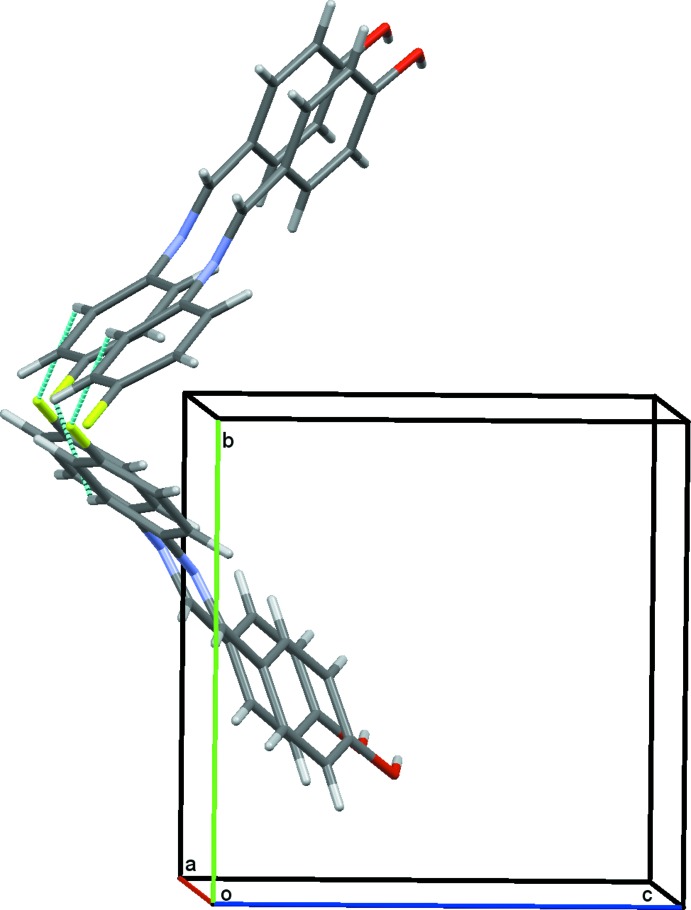
: C—H···F chains viewed along the a axis, showing V shaped stacks.

**Fig. 5.**
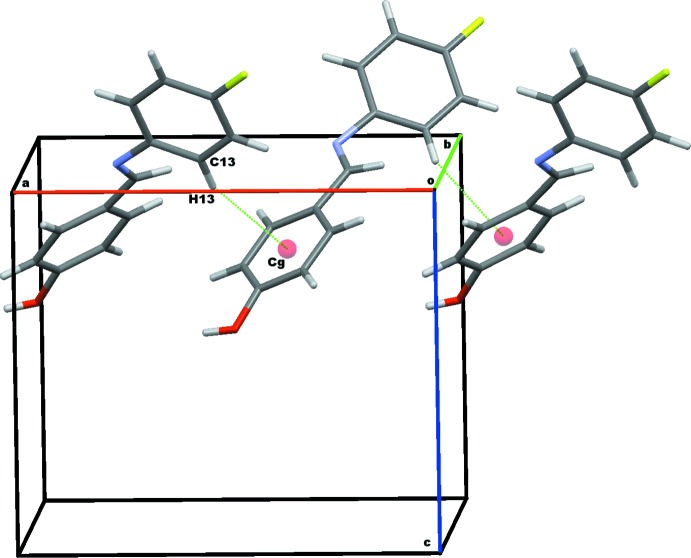
Chains formed along the a axis by C—H···π contacts.

**Fig. 6.**
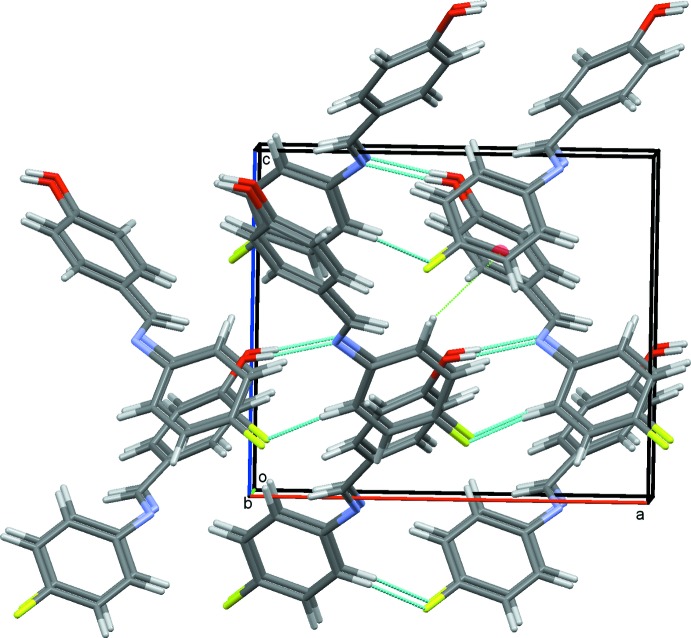
: Overall packing for the compound (I).

## supplementary crystallographic information

### S1. Comment

Schiff base compounds have been used as fine chemicals and pharmaceutical substrates (Akkurt *et al.*, 2013). They are important ligands in coordination chemistry due to their ease of preparation and ability to be modified both electronically and sterically (Li *et al.*, 2008 and Zhang, 2010). As a part of our study into the co-ordination behaviour of ligands having a 4-hydroxy substituent on the benzylidene fragment, X-ray structural analysis of the title compound was carried out, and the results are reported herein.

The title compound, (I), contains two benzene rings bridged by an HC ═N imine unit, with the planes of the rings inclined at an angle of 50.52 (8)°, showing significant deviation of the molecule from planarity as observed in the related structures 4-bromo-*N*-(4-hydroxybenzylidene)aniline and 4-[(*E*)-(4-methylphenyl)iminomethyl]phenol (Jothi *et al.*, 2012*a*,*b*). The molecule exists in the solid state in an *E*-configuration with respect to the C7 ═N1 double bond as indicated by the torsion angle C4–C7–N1–C8= -171.2 (2)°. The C4–C7 [1.456 (3) Å] and N1–C8 [1.430 (3) Å] distances confirm a degree of electron delocalization between the benzene rings, and the molecule can be regarded as a partially delocalized π-electron system. All other bond lengths are within the expected ranges (Allen *et al.*, 1987).

In the crystal, the molecules are linked by O1—H1···N1 hydrogen bonds to form infinite one-dimensional zigzag chains with graph set notation *C*(8) (Bernstein *et al.*,, 1995) along the *c* axis, Fig 2. Weaker C9—H9···F1 contacts also propagate *C*(5) zigzag chains along *b*, Fig 3, with molecules in this chain forming a V-shaped stacking motif when viewed along *a*, Fig 4. Finally C13—H13···π contacts also form chains along a, Fig 5. These contacts combine to stack the molecules in a head to tail zigzag fashion along the *b* axis direction, Fig 6.

### S2. Experimental

4-Fluoro-4-hydroxybenzylideneaniline was prepared by mixing equimolar amounts of 4-hydroxy benzaldehyde and 4-fluoro aniline in ethanol (40 ml). The reaction mixture was refluxed for about 6 h and the resulting solution was slowly evaporated at room temperature. After three days single crystals of the title compound, suitable for X-ray structure analysis were obtained.

### S3. Refinement

All the H atoms were positioned geometrically and treated as riding atoms: E—H = 0.93, 0.96, 0.97 and 0.82 Å for CH, CH_3_, CH_2_ and OH H atoms, respectively, with *U*_iso_(H) = k × *U*_eq_(C,*O*), where k = 1.5 for CH_3_ and OH H atoms and = 1.2 for other H atoms. The best crystal investigated was still of poor quality and very weakly diffracting, with no usable data obtained above θ = 23.5 °. Nonetheless the structure solved readily and refined to give acceptable uncertainties on the metrical data. Because of the very weak data, the final data/parameter ratio is considerably less than an ideal value.

### Crystal data

**Table:**

C_13_H_10_FNO	*F*(000) = 448
*M**_r_* = 215.22	*D*_x_ = 1.379 Mg m^−^^3^
Orthorhombic, *P**c**a*2_1_	Mo *K*α radiation, λ = 0.71073 Å
Hall symbol: P 2c -2ac	Cell parameters from 7057 reflections
*a* = 11.0153 (8) Å	θ = 1.9–23.4°
*b* = 9.8596 (7) Å	µ = 0.10 mm^−^^1^
*c* = 9.5476 (6) Å	*T* = 296 K
*V* = 1036.93 (12) Å^3^	Block, colourless
*Z* = 4	0.30 × 0.20 × 0.20 mm

### Data collection

**Table:**

Bruker KappaCCD APEXII diffractometer	1430 independent reflections
Radiation source: fine-focus sealed tube	1282 reflections with *I* > 2σ(*I*)
Graphite monochromator	*R*_int_ = 0.033
ω and φ scan	θ_max_ = 23.4°, θ_min_ = 2.8°
Absorption correction: multi-scan (*SADABS*; Bruker, 2004)	*h* = −12→12
*T*_min_ = 0.971, *T*_max_ = 0.980	*k* = −10→10
6612 measured reflections	*l* = −10→10

### Refinement

**Table:**

Refinement on *F*^2^	Primary atom site location: structure-invariant direct methods
Least-squares matrix: full	Secondary atom site location: difference Fourier map
*R*[*F*^2^ > 2σ(*F*^2^)] = 0.029	Hydrogen site location: inferred from neighbouring sites
*wR*(*F*^2^) = 0.078	H-atom parameters constrained
*S* = 1.11	*w* = 1/[σ^2^(*F*_o_^2^) + (0.0435*P*)^2^ + 0.103*P*] where *P* = (*F*_o_^2^ + 2*F*_c_^2^)/3
1430 reflections	(Δ/σ)_max_ < 0.001
146 parameters	Δρ_max_ = 0.13 e Å^−^^3^
1 restraint	Δρ_min_ = −0.12 e Å^−^^3^

### Special details

**Table:**

Geometry. All e.s.d.'s (except the e.s.d. in the dihedral angle between two l.s. planes) are estimated using the full covariance matrix. The cell e.s.d.'s are taken into account individually in the estimation of e.s.d.'s in distances, angles and torsion angles; correlations between e.s.d.'s in cell parameters are only used when they are defined by crystal symmetry. An approximate (isotropic) treatment of cell e.s.d.'s is used for estimating e.s.d.'s involving l.s. planes.
Refinement. Refinement of *F*^2^ against ALL reflections. The weighted *R*-factor *wR* and goodness of fit *S* are based on *F*^2^, conventional *R*-factors *R* are based on *F*, with *F* set to zero for negative *F*^2^. The threshold expression of *F*^2^ > σ(*F*^2^) is used only for calculating *R*-factors(gt) *etc*. and is not relevant to the choice of reflections for refinement. *R*-factors based on *F*^2^ are statistically about twice as large as those based on *F*, and *R*- factors based on ALL data will be even larger.

### Fractional atomic coordinates and isotropic or equivalent isotropic displacement parameters (Å^2^)

**Table:**

	*x*	*y*	*z*	*U*_iso_*/*U*_eq_	
C1	0.43955 (18)	0.3194 (2)	0.3175 (2)	0.0396 (6)	
C2	0.48448 (17)	0.44366 (19)	0.2735 (3)	0.0385 (5)	
H2	0.5578	0.4754	0.3091	0.046*	
C3	0.42173 (17)	0.5198 (2)	0.1781 (3)	0.0385 (5)	
H3	0.4526	0.6034	0.1502	0.046*	
C4	0.31198 (18)	0.4740 (2)	0.1219 (2)	0.0366 (5)	
C5	0.26905 (18)	0.3484 (2)	0.1656 (3)	0.0448 (6)	
H5	0.1963	0.3159	0.1295	0.054*	
C6	0.33162 (18)	0.2716 (2)	0.2608 (3)	0.0467 (6)	
H6	0.3018	0.1873	0.2875	0.056*	
C7	0.23844 (18)	0.5576 (2)	0.0292 (3)	0.0393 (5)	
H7	0.1598	0.5287	0.0100	0.047*	
C8	0.18716 (17)	0.7493 (2)	−0.1001 (3)	0.0371 (5)	
C9	0.2246 (2)	0.8203 (2)	−0.2169 (3)	0.0462 (6)	
H9	0.3051	0.8148	−0.2457	0.055*	
C10	0.1439 (2)	0.8992 (2)	−0.2914 (3)	0.0546 (6)	
H10	0.1687	0.9455	−0.3713	0.066*	
C11	0.0271 (2)	0.9080 (2)	−0.2453 (3)	0.0561 (7)	
C12	−0.0118 (2)	0.8445 (3)	−0.1267 (3)	0.0554 (7)	
H12	−0.0913	0.8554	−0.0957	0.067*	
C13	0.06838 (18)	0.7641 (2)	−0.0534 (3)	0.0470 (6)	
H13	0.0430	0.7196	0.0274	0.056*	
N1	0.27379 (14)	0.66729 (17)	−0.02753 (19)	0.0379 (4)	
O1	0.49408 (13)	0.24314 (15)	0.41734 (19)	0.0509 (4)	
H1	0.5637	0.2701	0.4294	0.076*	
F1	−0.05262 (16)	0.98467 (18)	−0.3178 (2)	0.0888 (6)	

### Atomic displacement parameters (Å^2^)

**Table:**

	*U*^11^	*U*^22^	*U*^33^	*U*^12^	*U*^13^	*U*^23^
C1	0.0291 (11)	0.0482 (13)	0.0415 (15)	0.0067 (10)	0.0037 (9)	−0.0017 (10)
C2	0.0253 (10)	0.0462 (12)	0.0441 (14)	−0.0021 (9)	−0.0006 (10)	−0.0035 (11)
C3	0.0299 (11)	0.0417 (11)	0.0438 (14)	−0.0023 (9)	0.0012 (11)	−0.0019 (10)
C4	0.0284 (11)	0.0433 (12)	0.0380 (14)	0.0013 (9)	0.0023 (9)	−0.0036 (10)
C5	0.0280 (11)	0.0515 (12)	0.0547 (16)	−0.0028 (9)	−0.0059 (10)	−0.0035 (12)
C6	0.0328 (12)	0.0468 (12)	0.0606 (17)	−0.0038 (9)	0.0004 (11)	0.0034 (12)
C7	0.0273 (10)	0.0473 (12)	0.0432 (14)	0.0004 (10)	−0.0030 (9)	−0.0087 (12)
C8	0.0312 (10)	0.0407 (11)	0.0395 (13)	0.0008 (9)	−0.0042 (10)	−0.0055 (10)
C9	0.0421 (12)	0.0463 (12)	0.0503 (16)	−0.0015 (10)	0.0065 (11)	−0.0027 (12)
C10	0.0680 (17)	0.0474 (13)	0.0483 (17)	0.0061 (11)	0.0002 (13)	0.0047 (12)
C11	0.0619 (16)	0.0514 (14)	0.0549 (18)	0.0215 (12)	−0.0119 (13)	−0.0033 (13)
C12	0.0403 (12)	0.0686 (16)	0.0574 (18)	0.0146 (12)	−0.0034 (12)	−0.0081 (14)
C13	0.0367 (12)	0.0595 (14)	0.0447 (16)	0.0075 (11)	0.0020 (10)	0.0027 (11)
N1	0.0297 (8)	0.0458 (9)	0.0383 (11)	0.0018 (8)	0.0003 (8)	−0.0037 (9)
O1	0.0353 (7)	0.0614 (9)	0.0561 (11)	−0.0015 (8)	−0.0049 (7)	0.0140 (9)
F1	0.0971 (12)	0.0911 (11)	0.0781 (12)	0.0487 (10)	−0.0175 (10)	0.0104 (10)

### Geometric parameters (Å, º)

**Table:**

C1—O1	1.355 (3)	C8—C9	1.379 (3)
C1—C2	1.386 (3)	C8—C13	1.390 (3)
C1—C6	1.389 (3)	C8—N1	1.430 (3)
C2—C3	1.368 (3)	C9—C10	1.379 (3)
C2—H2	0.9300	C9—H9	0.9300
C3—C4	1.398 (3)	C10—C11	1.362 (3)
C3—H3	0.9300	C10—H10	0.9300
C4—C5	1.389 (3)	C11—F1	1.350 (3)
C4—C7	1.456 (3)	C11—C12	1.363 (4)
C5—C6	1.369 (3)	C12—C13	1.378 (3)
C5—H5	0.9300	C12—H12	0.9300
C6—H6	0.9300	C13—H13	0.9300
C7—N1	1.270 (3)	O1—H1	0.8200
C7—H7	0.9300		
			
O1—C1—C2	123.07 (19)	C9—C8—C13	119.2 (2)
O1—C1—C6	117.74 (19)	C9—C8—N1	118.62 (18)
C2—C1—C6	119.2 (2)	C13—C8—N1	122.1 (2)
C3—C2—C1	120.44 (19)	C10—C9—C8	120.7 (2)
C3—C2—H2	119.8	C10—C9—H9	119.7
C1—C2—H2	119.8	C8—C9—H9	119.7
C2—C3—C4	121.00 (19)	C11—C10—C9	118.6 (2)
C2—C3—H3	119.5	C11—C10—H10	120.7
C4—C3—H3	119.5	C9—C10—H10	120.7
C5—C4—C3	117.9 (2)	F1—C11—C10	119.0 (3)
C5—C4—C7	119.87 (18)	F1—C11—C12	118.6 (2)
C3—C4—C7	122.09 (18)	C10—C11—C12	122.4 (2)
C6—C5—C4	121.40 (19)	C11—C12—C13	119.0 (2)
C6—C5—H5	119.3	C11—C12—H12	120.5
C4—C5—H5	119.3	C13—C12—H12	120.5
C5—C6—C1	120.1 (2)	C12—C13—C8	120.1 (2)
C5—C6—H6	119.9	C12—C13—H13	120.0
C1—C6—H6	119.9	C8—C13—H13	120.0
N1—C7—C4	124.80 (18)	C7—N1—C8	118.89 (16)
N1—C7—H7	117.6	C1—O1—H1	109.5
C4—C7—H7	117.6		
			
O1—C1—C2—C3	−176.2 (2)	N1—C8—C9—C10	179.1 (2)
C6—C1—C2—C3	1.7 (3)	C8—C9—C10—C11	1.5 (3)
C1—C2—C3—C4	−0.7 (3)	C9—C10—C11—F1	−179.8 (2)
C2—C3—C4—C5	−0.3 (3)	C9—C10—C11—C12	1.7 (4)
C2—C3—C4—C7	174.8 (2)	F1—C11—C12—C13	178.8 (2)
C3—C4—C5—C6	0.1 (3)	C10—C11—C12—C13	−2.6 (4)
C7—C4—C5—C6	−175.1 (2)	C11—C12—C13—C8	0.4 (4)
C4—C5—C6—C1	1.0 (4)	C9—C8—C13—C12	2.6 (3)
O1—C1—C6—C5	176.2 (2)	N1—C8—C13—C12	179.8 (2)
C2—C1—C6—C5	−1.9 (3)	C4—C7—N1—C8	−171.2 (2)
C5—C4—C7—N1	−172.8 (2)	C9—C8—N1—C7	−145.9 (2)
C3—C4—C7—N1	12.2 (3)	C13—C8—N1—C7	36.9 (3)
C13—C8—C9—C10	−3.6 (3)		

### Hydrogen-bond geometry (Å, º)

Cg is the centroid of the C1–C6 benzene ring.

**Table:**

*D*—H···*A*	*D*—H	H···*A*	*D*···*A*	*D*—H···*A*
O1—H1···N1^i^	0.82	1.94	2.756 (2)	176
C9—H9···F1^ii^	0.93	2.61	3.263 (3)	127
C13—H13···*Cg*^iii^	0.93	2.83	3.710 (3)	157

Symmetry codes: (i) −*x*+1, −*y*+1, *z*+1/2; (ii) *x*+1/2, −*y*+2, *z*; (iii) *x*−1/2, −*y*+1, *z*.
